# Effects of Total Dietary Fiber on Cecal Microbial Community and Intestinal Morphology of Growing White Pekin Duck

**DOI:** 10.3389/fmicb.2021.727200

**Published:** 2021-09-01

**Authors:** Yongsheng Hao, Zhanqing Ji, Zhongjian Shen, Yongbao Wu, Bo Zhang, Jing Tang, Shuisheng Hou, Ming Xie

**Affiliations:** State Key Laboratory of Animal Nutrition, Institute of Animal Science, Chinese Academy of Agricultural Sciences, Beijing, China

**Keywords:** total dietary fiber, ducks, microbiota, short-chain fatty acids, barrier function

## Abstract

The current study was to investigate the effects of total dietary fiber (TDF) on growth performance, cecal structure, cecal microbial community, and short-chain fatty acids (SCFAs) profiles in the cecum of growing White Pekin ducks. A total of 108 male Pekin ducks of 14-days-old were randomly allocated and fed diets containing 12.4, 14.7, and 16.2% TDF for 35 days. Each dietary treatment consisted of six replicates with six birds each. The results showed that 14.7 and 16.2% TDF treatments promoted growth performance relative to 12.4% TDF treatments (*P* < 0.05). A total of 14.7 and 16.2% TDF treatments significantly elevated villus height, the ratio of villus height to crypt depth and muscle layer thickness of cecum, and lowered crypt depth compared with 12.4% TDF treatment (*P* < 0.05). Simultaneously, 14.7 and 16.2% TDF treatments up-regulated *Claudin-1* mRNA expression of barrier genes in the cecum compared with 12.4% TDF (*P* < 0.05). Butyrate-producing bacteria like Oscillopiraceae affiliating to the phyla Firmicutes were observed as a biomarker in the 16.2% TDF. Higher concentration of butyrate in the cecum was obtained in the 14.7% TDF compared with 12.4 and 16.2% TDF (*P* < 0.05). The concentrations of isobutyrate, valerate, and isovalerate in the cecum were significantly increased in the 16.2% TDF compared with 12.4 and 14.7% TDF (*P* < 0.05). Meanwhile, the abundance of genus *UCG-005* and *Enterococcus* was positive correlations with isobutyrate and valerate (*P* < 0.05). However, the concentration of propionate in the cecum significantly decreased in 14.7 and 16.2% TDF treatments relative to 12.4% TDF treatments (*P* < 0.05). In summary, increasing TDF levels improved growth performance, cecal histomorphology, and barrier function of meat ducks and it might be mediated by the changes of microbiota communities, especially bloom of SCFAs-producing bacteria, which facilitated the interaction between intestinal mucosa and microbiota.

## Introduction

Nowadays, use of antibiotics has been banned in some countries and gut barrier health is becoming a potential risk for poultry production. Fortunately, the beneficent role of fibers in modulating gut microbiome, stimulating immunity, and promoting gut integrity is noticed (Singh and Kim, 2021; Tejeda and Kim, 2021) and inclusion of fibers in poultry diets is becoming a nutritional strategy to protect poultry gut from invasion of pathogen and toxins (Kheravii et al., 2018), although the fiber contents from feedstuffs had lowering-digestibility and antinutrient properties. Likes other avian species, dietary fiber was also beneficial to duck growth and gut health and ducks could adapt to a wide dietary crude fiber range from 3.09 to 7.52% (Han et al., 2017). Furthermore, ducks fed with high dietary crude fiber levels had increased gizzard development, jejunal morphology, energy retention, excreta nutrients availability, and standardized ileal digestibility of amino acids, and high dietary fiber also alleviated hepatic fat deposition via inhibiting lipogenic gene expression in meat ducks (Han et al., 2017; Qin et al., 2018).

In the past decades, the concept of crude fiber has still been commonly used in poultry diet formulation, but this concept may be questioned because non-starch polysaccharides and resistant starch are usually ignored by this concept, and these ignored fibrous compounds play a crucial role in intestinal functioning, nutrient digestion, and intestinal microflora modulation. At the same crude fiber levels, the starter broilers fed fiber from soy hulls had better growth performance, intestinal histomorphology, and nutrient digestibility compared with the birds fed fiber from purified cellulose (Tejeda and Kim, 2020). Recently, when ducks were fed with diets containing high levels of resistant starch, the gut barrier was improved by enhancing intestinal morphology and barrier markers expression, modulating the microbiota composition, and attenuating inflammatory markers (Qin et al., 2019, 2020). In the aforementioned two studies, soy hulls contained many fibrous components out of category of crude fiber, and resistant starch was out of category of crude fiber. Fortunately, compared with the concept of crude fiber, the more fiber fractions including non-starch polysaccharides and resistant starch were covered by the concept of total dietary fiber (TDF) developed by the Association of Official Analytical Chemists, AOAC International, 2007, and thus the concept of total crude fiber may be preferable to the concept of crude fiber when the relationship between dietary fiber and gut health was emphasized.

Recently, the association between microbial community and ileal gene expression on intestinal wall thickness alterations in chickens revealed that well-developed intestinal morphology could increase the abundance of beneficial bacteria coupled with active community anabolism, thus enhancing the absorption and immune function of intestinal epithelial cells (Tang et al., 2020). Furthermore, the microbial community and short-chain fatty acids (SCFAs) mapping in the intestinal tract of quail showed that cecum was the core location of fiber fermentation and SCFAs production because cecum has the most total bacteria population and highest SCFAs concentrations among all intestinal segments (Du et al., 2020). When resistant starch was fed to ducks, the ducks with improved cecal morphology also had enhanced the abundance of SCFAs-producing bacteria in the cecum (Qin et al., 2020). In pigs, the positive effects of dietary fiber on intestinal barrier function also could be explained by improving distal gut morphology and altering microbiota composition, especially enhancing the abundance of butyrate-producing bacteria (Chen et al., 2013). Recently, the concept of TDF was used in pig diet formulation, and appropriate TDF level could increase the diversity and metabolic capacity of cecal microbiota to improve the utilization efficiency of fiber resources without altering the growth rate of pigs (Pu et al., 2020). However, in ducks, the researches on the potential beneficial effects of TDF on growth performance and gut health were still limited, and it was not clear for gut–microbiota interplay at high fiber intake. Therefore, the concept of TDF was utilized in the present study and the objective of our study was to discuss the effects of TDF on growth performance, cecal histomorphology, and cecal microbiota of growing Pekin ducks.

## Materials and Methods

### Experimental Design and Bird Management

The dose-response experiment with three TDF levels (12.4, 14.7, and 16.2%) was conducted with 14-days-old male White Pekin ducks. A total of 120 one-day-old male white Pekin ducklings from one commercial hatchery were fed with a commercial starter diet containing 12.12 MJ metabolizable energy/kg and 200 g crude protein/kg of diet until 14 days of age. At 14 days of age, all birds were weighed individually and the birds with the lowest or highest body weight were removed, and 108 birds were selected from the remaining birds. Afterward, these ducks were allotted to 18 cages of 6 birds according to similar cage weight. Each dietary treatment had six replicate cages. The experimental diets were fed from 14 to 35 days of age. All ducks were given free access to water and feed and lighting was continuous. The temperature was kept at 33°C from 1 to 3 days of age and then it was reduced gradually to approximately 25°C until 14 days of age and was kept at approximately 16 to 22°C during the growing period from 14 to 35 days of age.

### Experimental Diets

Experimental diets with low, medium, and high TDF levels were formulated and the feed composition of all these diets are provided in Table 1. All experimental diets were cold-pelleted at room temperature and the TDF levels of these diets also were analyzed according to the method of AOAC International (2007). The analyzed TDF levels of these 3 experimental diets were 12.4, 14.7, and 16.2%, respectively.

**TABLE 1:** Ingredient and composition of the experimental diets (%, dry matter basis).

Item	Content (%)		
	
	Low TDF diet	Medium TDF diet	High TDF diet
Corn	66.36	66.49	66.62
Soybean meal	28.10	14.05	0.00
Isolated soybean protein	0.00	6.00	12.00
Soybean dietary fiber	0.00	6.35	12.70
Soy oil	1.50	2.75	4.00
DL-Methionine	0.14	0.175	0.21
L-Lysine-HCl	0.00	0.035	0.07
L-Threonine	0.00	0.065	0.13
L-Tryptophan	0.00	0.035	0.07
Dicalcium phosphate	1.50	1.55	1.60
Limestone	1.10	1.20	1.30
Salt	0.30	0.30	0.30
Vitamin-mineral premix^a^	1.00	1.00	1.00
**Calculated nutrient levels**			
ME (Mcal/kg)	2.92	2.91	2.90
Crude protein, %	17.67	17.685	17.70
Lysine, %	0.90	0.885	0.87
Methionine, %	0.41	0.41	0.41
Methionine + cystine, %	0.70	0.63	0.56
Tryptophan, %	0.22	0.215	0.21
Threonine, %	0.73	0.725	0.72
Calcium, %	0.80	0.80	0.80
Total phosphorus, %	0.61	0.58	0.55
Non-ohytate phosphorus, %	0.39	0.39	0.39
**Analyzed value**			
TDF, %	12.4	14.7	16.2

*^*a*^Supplied per kilogram of total diet: Cu, 8 mg; Fe, 60 mg; Zn, 60 mg; Mn, 100 mg; Se, 0.3 mg; I, 0.4 mg; choline chloride, 1,000 mg; vitamin A, 4,000 IU; vitamin D3, 2,000 IU; vitamin E, 20 IU; vitamin K3, 2 mg; thiamin, 2 mg; riboflavin, 10 mg; pyridoxine hydrochloride, 4 mg; cobalamin, 0.02 mg; calcium-D-pantothenate, 20 mg; nicotinic acid, 50 mg; folic acid, 1 mg; and biotin, 0.15 mg. TDF, total dietary fiber.*

### Sample Collection

At 35 days of age, the body weight (BW), average daily gain (ADG), average daily feed intake (ADFI), and feed to gain ratio (F/G) of ducks from each cage were measured. Feed intake and F/G were all corrected for mortality. Afterward, two ducks were randomly selected from each cage and euthanized by CO_2_ inhalation. The two ceca of each selected ducks were collected. Cecal content was collected from one cecum for microbiota and SCFAs analysis, and mucosa was scraped by sterile blade after saline flush for gene expression analysis, and both of them were stored at −80°C. One centimeter in length of the other cecum was collected and fixed in 4% paraformaldehyde solution for gut morphology analysis.

### Gut Morphology

The samples of cecum fixed in 4% paraformaldehyde solution were embedded in paraffin, and then sectioned at 5 μm and stained with hematoxylin and eosin using the standard procedures. The sections were pictured by Sony Alpha6000 APS camera. Then villus height (VH), crypt depth (CD), and muscle layer thickness (MLT) of cecum were analyzed by Image Pro-Plus 6.0 software (Media Cybernetics, Bethesda, MD, United States) at 100× magnification.

### SCFAs Analysis

Cecal content was weighted approximately 0.5 g and diluted with 2 ml ultrapure water, tempestuously commixed and centrifuged (10,000 *g*, 15 min at 4°C) following 900 μl supernatant being mixed with prepared 100 μl ice-cold 25% (w/v) metaphosphoric acid solution at 4°C for 4 h in a shaded environment. Then the mixture was centrifuged (10,000 *g*, 15 min at 4°C) and the solution was filtered with 45 μm nylon microporous membrane by syringe. The gas chromatography system measured concentrations of acetate, propionate, isobutyrate, butyrate, isovalerate, and valerate by DB-FFAP column (30 m × 250 μm × 0.25 μm). The carrier gas was the N_2_ (12.5 Mpa, 0.8 ml/min). The temperature of FID detector was 280°C and that of column heated from 60 to 220°C at a rate of 20°C/min.

### Cecal Microbiota Analysis

Total genomic DNA from cecal content samples was extracted by QIAamp DNA Stool Mini Kit (Qiagen, Hilden, Germany) according to the manufacturer’s instructions. DNA concentration and integrality were detected by NanoDrop Spectrophotometer (Thermo Fisher Scientific, Wilmington, DE, United States) and agarose gel electrophoresis, respectively. DNA concentration of each cecal content was diluted to 10 ng/μl using double-distilled water. The V3–V4 region of 16S rDNA was amplified using the following specific primers (338F:5′-ACTCCTACGGGAGGCAGCAG-3′; 806R: 5′-GGACTACHVGGGTWTCTAAT-3′). Purified amplicons were pooled in equal amounts and paired-end sequenced (2 × 250 bp) on an Illumina MiSeq platform at Majorbio Bio-Pharm Technology Co., Ltd. (Shanghai, China).

### Gene Expression

Total RNA from cecal mucous membrane was extracted by TRIzol reagent (Takara), according to the manufacturer’s instruction book. Primer premier 6 was used to custom primers that are shown in Supplementary Table 1. The number of 1,000 ng of total RNA was applied to synthesized cDNA using the PrimeScript RT Reagent Kit (Takara). ABI 7500 Real-time PCR Instrument implemented Real-time PCR (Applied Biosystems). PCR system was comprised of 1 μl of five times diluted cDNA, 0.4 μl each of 10 μM forward and reverse primers, 5 μl TB Green Premix Ex Taq II (Takara), 0.2 μl ROX Reference Dye II (Takara) and 3 μl DNase Free dH_2_O. The PCR amplification system was in two stages including 95°C for 30 s, followed by 40 cycles both of 95°C for 5 s and 60°C for 34 s. The specificity of the primers was examined by a melting curve analysis. The gene β*-actin* was selected as a reference gene, which was used to normalize the relative expression of genes of interest by the 2^–ΔΔ*CT*^ method.

### Statistical Analysis

The data in a completely randomized design were analyzed using the one-way ANOVA procedure of SAS 9.4 software (SAS Institute, Inc.), with cage as the experimental unit for analyzing growth performance and each selected bird as the experimental unit for other parameters. *P* < 0.05 was considered statistical significance. All data were expressed as means and pooled SEM.

For sequence processing, the raw reads were demultiplexed and quality-filtered by QIIME pipeline (version 1.17) (Edgar, 2010). Quality-filtered was performed to filter low-quality reads with average Phred scores lower than 20 and potential chimeric sequences were discarded using Uchime algorithm (Edgar et al., 2011). Then FLASH was used to merge reads (Magoc and Salzberg, 2011). The available sequences were clustered into operational taxonomic units (OTUs) according to 97% similarity. Taxonomy of OTUs were performed using the SILVA database. For α-diversity analysis and β-diversity using principal coordinate analysis (PCoA) based on unweighted Unifrac distance, R package “vegan” and “ggplot2” were implemented to plot the results. The significant distinction of microbial communities was estimated by ANOSIM based on unweighted Unifrac distance with R package “vegan.” The relative abundance of bacteria at family level was analyzed by one-way ANOVA among groups and Welch’s *t*-test was applied to *post hoc* using R package “stats” and Python package “scipy.” The different abundance of microbiota communities from phylum to genus was analyzed using the linear discriminant analysis (LDA) effect size (LEfSe) algorithm under the non-parametric factorial Kruskal–Wallis sum-rank test with *P* < 0.05. LDA score (>2) was to examine significant microbiota communities. Spearman correlation was used to analyze the relationship between microbial community taxa and variable factors including acetate, propionate, isobutyrate, butyrate, isovalerate, and valerate using R package “pheatmap” with *P* < 0.05. Linear regression was devised to evaluate collinearity of variable factors using variance inflation factor (VIF) (<10) with R package “tidyverse” and “caret.”

## Results

### Growth Performance

The effects of TDF on growth performance of White Pekin ducks are exhibited in Table 2. Compared with diets containing 12.4 and 14.7% TDF, the 16.2% TDF diet had distinctly ameliorated (*P* < 0.05) the BW of birds at 35 days. The ADG of birds significantly increased (*P* < 0.05) with increasing dietary fiber levels. The 16.2% TDF treatments significantly lowered (*P* < 0.05) F/G of birds compared with the experimental treatments containing 12.4 and 14.7% TDF.

**TABLE 2:** Effects of total dietary fiber on growth performance of White Peking ducks on day 35.

Item	12.4% TDF	14.7% TDF	16.2% TDF	SEM	*P-*value
BW (g)	2396^b^	2461^b^	2533^a^	23.4	<0.01
ADG (g)	87.0^b^	90.0^ab^	93.4^a^	1.54	<0.01
ADFI (g)	192	196	193	2.39	0.624
F/G	2.21^b^	2.17^b^	2.07^a^	0.02	<0.01

*^*a,b*^Means with different superscripts in the same row differ significantly (*n* = 6; *P* < 0.05). 12.4% TDF, 12.4% total dietary fiber; 14.7% TDF, 14.7% total dietary fiber; 16.2% TDF, 16.2% total dietary fiber; BW, body weight; ADG, average daily gain; ADFI, average daily feed intake; F/G, the ratio of feed to gain.*

### Cecal Morphology

The parameters of sections of cecal morphology are shown in Table 3. The birds fed 14.7 and 16.2% TDF diets had significantly improved (*P* < 0.05) VH compared with other birds fed 12.4% TDF diet. The 14.7 and 16.2% TDF treatments significantly lowered (*P* < 0.05) the CD of birds. The V/C and MLT in the cecum of birds were dramatically increased (*P* < 0.05) when birds fed 14.7% TDF diet.

**TABLE 3:** Effects of total dietary fiber on cecal morphology of White Peking ducks on day 35.

Item	12.4% TDF	14.7% TDF	16.2% TDF	SEM	*P-*value
VH (μm)	487^b^	622^a^	646^a^	16.9	<0.01
CD (μm)	123^b^	100^a^	105^a^	3.34	<0.01
V/C	3.99^b^	6.26^a^	6.21^a^	0.230	<0.01
MLT (μm)	281^*c*^	359^a^	330^b^	8.99	<0.01

*^*a*–*c*^Means with different superscripts in the same row differ significantly (*n* = 6; *P* < 0.05). 12.4% TDF, 12.4% total dietary fiber; 14.7% TDF, 14.7% total dietary fiber; 16.2% TDF, 16.2% total dietary fiber; VH, villus height; CD, crypt depth; V/C, the ratio of VH to CD; MLT, muscle layer thickness.*

### SCFAs Profiling

**FIGURE 1:**
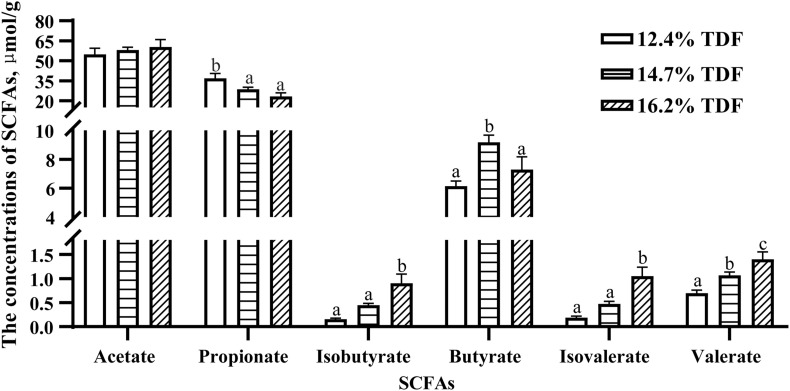
Effects of total dietary fiber on the concentrations of cecal SCFAs of White Peking ducks on day 35. ^a– *c*^Means with different superscripts in the same index differ significantly (*n* = 12, *P* < 0.05). 12.4% TDF, 12.4% total dietary fiber; 14.7% TDF, 14.7% total dietary fiber; 16.2% TDF, 16.2% total dietary fiber; SCFAs, short-chain fatty acids.

The concentrations of SCFAs were measured and are shown in Figure 1. The concentration of propionate was higher (*P* < 0.05) in the cecum of ducks fed 12.4% TDF diet compared with other experimental diets. Butyrate concentration was conspicuous (*P* < 0.05) in the diets containing 14.7% TDF compared with 12.4 and 16.2% TDF diets. The concentrations of isobutyrate, isovalerate, and valerate were significantly increased (*P* < 0.05) in the 16.2% TDF diet relative to 12.4 and 16.2% TDF diets.

### Cecal Microbial Analysis

**FIGURE 2:**
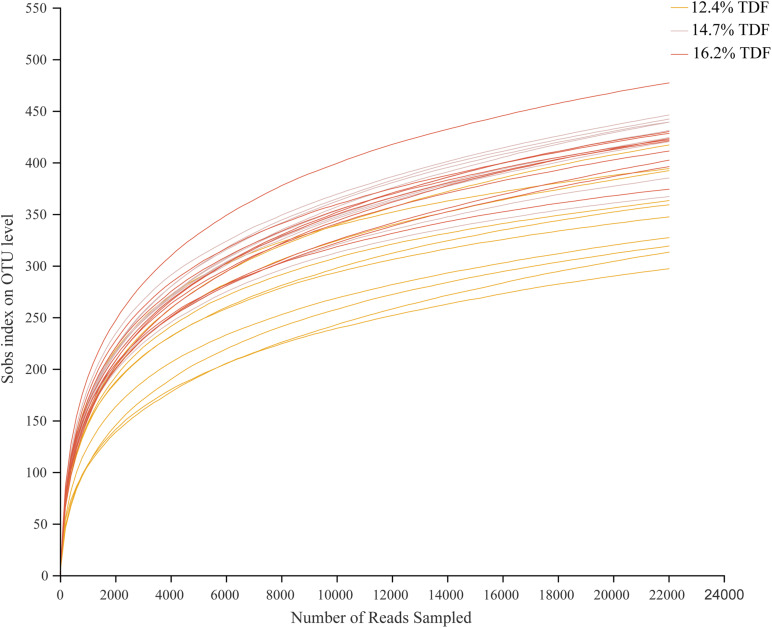
Effects of total dietary fiber on cecal microbial rarefaction curve of White Pekin ducks on day 35. 12.4% TDF, 12.4% total dietary fiber; 14.7% TDF, 14.7% total dietary fiber; 16.2% TDF, 16.2% total dietary fiber.

**FIGURE 3:**
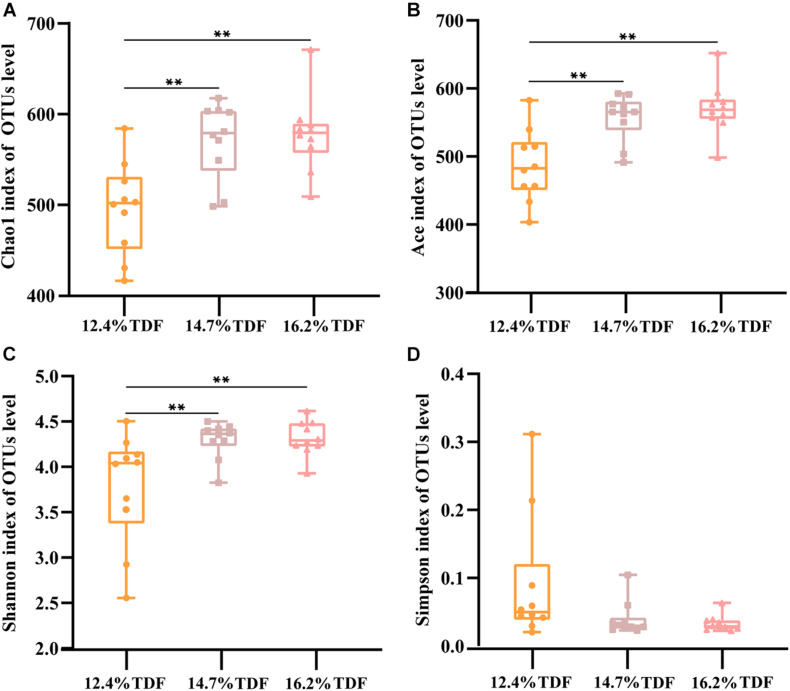
Effects of total dietary fiber on cecal microbial diversity of White Peking ducks on day 35. Significant difference was recorded by 0.01 < *P* ≤ 0.05^∗^, 0.001 < *P* ≤ 0.01^∗∗^, *P* ≤ 0.001^∗∗∗^. 12.4% TDF, 12.4% total dietary fiber; 14.7% TDF, 14.7% total dietary fiber; 16.2% TDF, 16.2% total dietary fiber.

**FIGURE 4:**
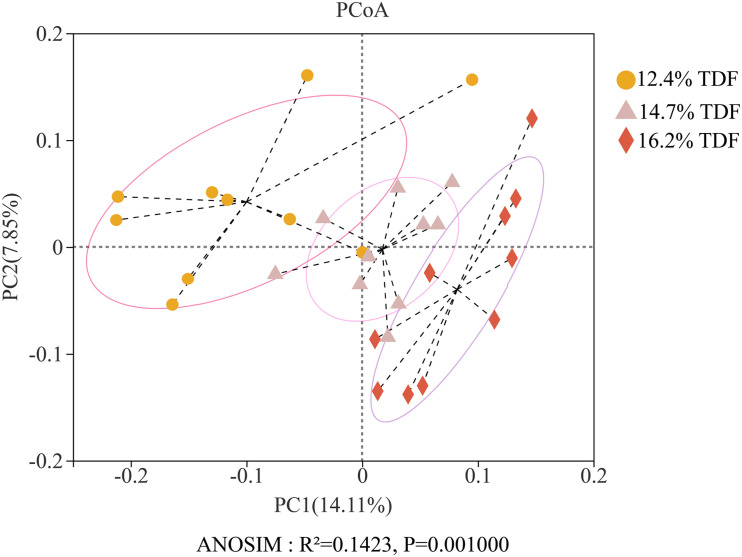
Effects of total dietary fiber on β-diversity based on unweighted Unifrac distance calculated from OTUs abundance matrix of White Pekin ducks on day 35.

After quality-filtered and merge, the average of 22,036 available sequences of each sample was generated, and 1,010 OTUs were obtained. Rarefaction curves analysis based on sobs index showed sample sequencing depths were sufficient to cover almost all microbes in the samples (Figure 2). Compared with 12.4% TDF groups, the α-diversity estimators (Figure 3) of richness including ace and chao1 were significantly increased (*P* < 0.05) in 14.7 and 16.2% TDF groups, and that of diversity assessed by Shannon were conspicuously raised (*P* < 0.05). Microbial profile was clustered using PCoA (Figure 4) based on unweighted Unifrac distance, by which cecal microbial communities of ducks fed three levels of dietary fiber diet were distributed three detached clusters. ANOSIM method based on unweighted Unifrac distance ulteriorly illustrated that microbial communities was the significant distinction in different content of dietary fiber groups (*R*^2^ = 0.336, *P* < 0.05) indicating that increasing dietary fiber exerted an effect on microbial composition.

**FIGURE 5:**
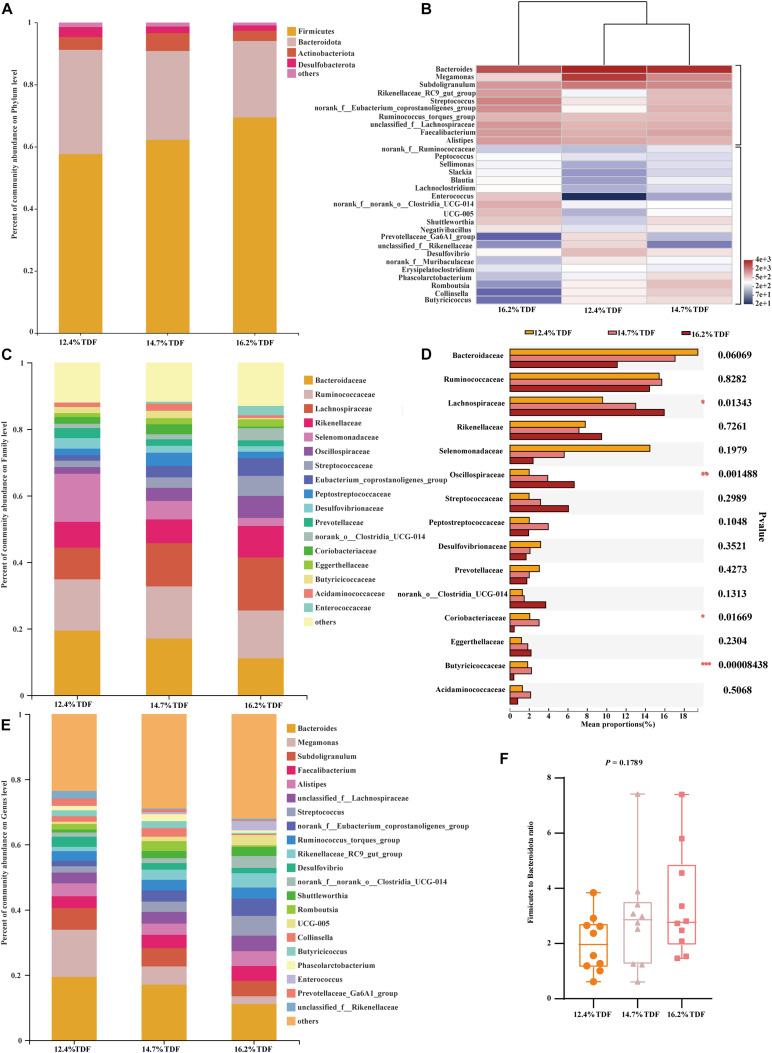
Effects of total dietary fiber on composition of cecal microbiota and differential species identified at phylum, family and genus level of White Pekin ducks on day 35. **(A,C,E)** were microbiota composition at phylum, family, and genus level, respectively; **(B)** was the heatmap of top 30 relative abundance genus of microbiota communities; **(D)** was differential bacteria at family level and **(F)** was the ratio of the abundance of Firmicutes to Bacteroidetes at phylum level. Significant difference was recorded by 0.01 < *P* ≤ 0.05*, 0.001 < *P* ≤ 0.01**, *P* ≤ 0.001***.

In order to analyze microbial composition, phyla, family, and genus were selected as taxonomic levels. Firmicutes, Bacteroidota, Actinobacteriota, and Desulfobacterota were the main phyla (>1%) in the cecum of duck, and Firmicutes and Bacteroidota were the dominant phyla while the relative abundance of Firmicutes was higher than that of Bacteroidota in all groups (Figure 5A). At genus level, the heatmap (Figure 5B) of top 30 most relative abundance genus showed cecal microbial communities of duck fed 12.4% and 14.7% TDF diets formed a common cluster while that of duck fed 16.2% TDF diet structured a separate cluster indicating microbial composition was similar between 12.4% TDF group and 14.7% TDF group. In addition, cecal microbiota were mainly clustered into two groups according to the relative abundance (Figure 5B), which one of them had higher relative abundance including *Bacteroides* and *Megamonas*. The duck’s cecal microbial communities of three groups was predominated by sequences representative of *Bacteroidaceae*, *Ruminococcaceae* and *Lachnospiraceae* at family level (Figure 5C). Compared with 12.4% TDF group, the relative abundance of *Lachnospiraceae*, *Oscillospiraceae* and *Coriobacteriacea* at family level was distinctly ameliorated (*P* < 0.05) after ducks fed 16.2% TDF diet. Besides, the relative abundance of *Butyricicoccacea* was in a significant rise (*P* < 0.05) in the 14.7% TDF group at family level (Figure 5D). *Bacterodies* and *Megamonas* were prominent genus in the cecum of duck, but the relative of Megamonas was lowered with increasing TDF level (Figure 5E). Firmicutes to Bacteroidota ratio was raised in the higher dietary fiber diet (Figure 5F).

**FIGURE 6:**
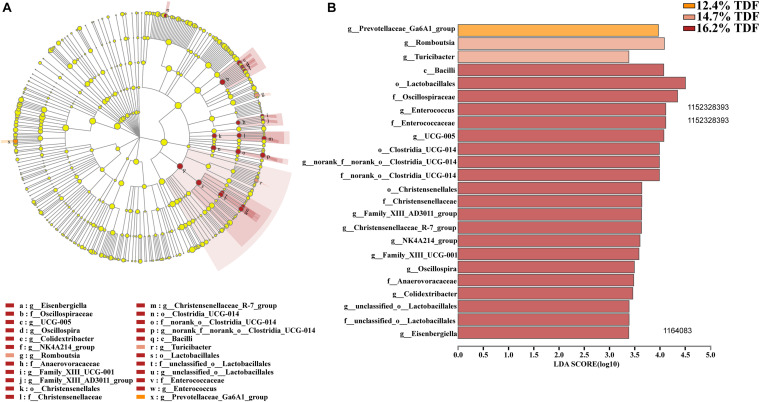
Effects of total dietary fiber on Linear discriminant analysis effect size (LEfSe) to detect the most significantly abundant cecal microbiota of White Pekin ducks on day 35 among three groups. **(A)** cladoram measured from LEfSe analysis; **(B)** LDA score generated for differentially abundant microbiota (LDA > 2, *P* < 0.05).

Furthermore, LEfSe analysis (Figure 6) was explored to identify significant taxa in phylotypes. In the aggregate, 14 genera were detected with LDA threshold >2. Ducks that were fed 16.2% TDF diet had significantly enriched (*P* < 0.05) *Bacilli* relative abundance at class level. The relative abundance of Anaerovoracaceae, Oscillospiraceae, Christensenellaceae, Enterococcaceae, *Norank_o_clostridia_UCG_014*, and *unclassified_o_Lactobacillales* at at family level was increased (*P* < 0.05) compared with ducks fed lower TDF diets. Moreover, the genus *Turicibacter* and *Romboutsia* were biomarkers in the 14.7% TDF group. Besides, the cecal microbial community of ducks was characterized (*P* < 0.05) by *Prevotellaceae_Ga6A1_group* as a biomarker when ducks were fed 12.4% TDF diet.

### Correlations Between SCFAs and Microbiota

**FIGURE 7:**
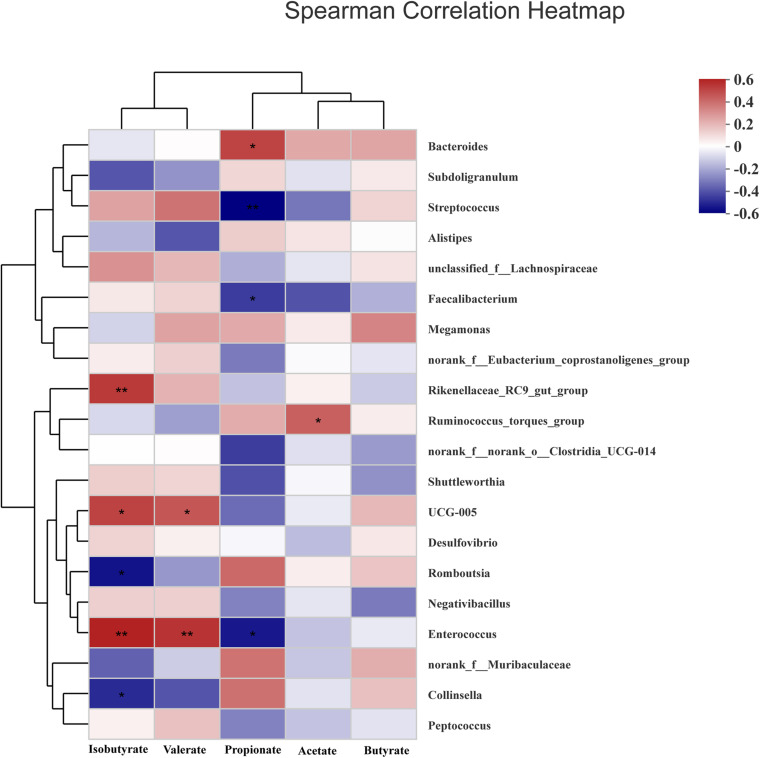
Heatmap of spearman’s correlation between cecal microbiota at genus level and SCFAs including acetate, propionate, butyrate, isobutyrate, and valerate in the cecum of White Pekin ducks on day 35. Significant correlation was recorded by 0.01 < *P* ≤ 0.05*, 0.001 < *P* ≤ 0.01**, *P* ≤ 0.001***.

To analyze microbiota associated with SCFAs as its metabolites, spearman analysis (Figure 7) was taken to evaluate the associations between SCFAs with VIF < 10 in the cecum of ducks and top 20 most abundant genus. The concentrations of acetate and propionate were positively associated with *Ruminococcus_torques_group* (*P* < 0.05) and *Bacteroides* (*P* < 0.05) respectively, whereas propionate was negatively linked with *Streptococcus*, *Enterococcus*, and *Faecalibacterium* (*P* < 0.05). Isobutyrate and valerate had a significantly positive correlation with *UCG-005* and *Enterococcus* (*P* < 0.05) while *Romboutsia* and *Collinsella* presented a negative correlation with isobutyrate (*P* < 0.05).

### Gene Expression

**FIGURE 8:**
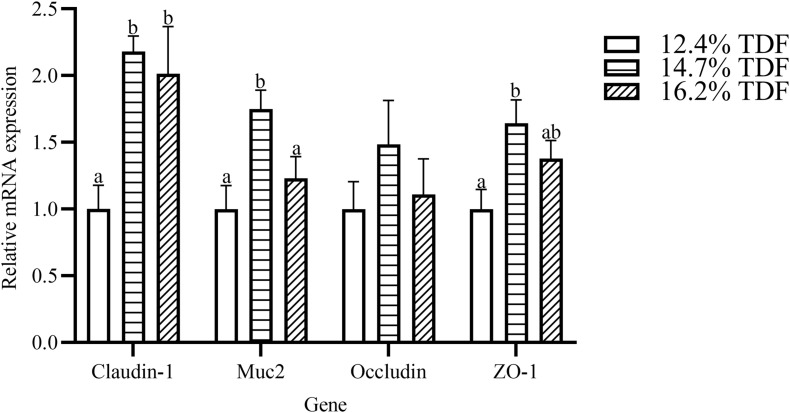
Effects of total dietary fiber on the relative mRNA expression of cecal barrier function genes of White Pekin Ducks on day 35. ^*a,b*^Means with different superscripts in the same index differ significantly (*n* = 6, *P* < 0.05). 12.4% TDF, 12.4% total dietary fiber; 14.7% TDF, 14.7% total dietary fiber; 16.2% TDF, 16.2% total dietary fiber; Muc2, Mucin-2; ZO-1, Zonula occludens-1.

The effects of TDF on cecum barrier function were investigated and the relative mRNA expression of intestinal barrier genes including *Zonula occludens-1* (*ZO-1*), *Mucin-2* (*Muc2*), *Occludin*, and *Claudin-1* was shown in Figure 8. The expression of *Claudin-1* gene was distinctly raised (*P* < 0.05) in the cecum of ducks supplemented with 14.7 and 16.2% TDF diets. When compared with the 12.7% TDF, the 14.7% TDF diets significantly up-regulated (*P* < 0.05) the expression of *Muc2* and *ZO-1* genes.

## Discussion

In our study, increasing TDF levels could promote duck growth by increasing weight gain, and it was accompanied with the markedly improvement in cecal morphology and barrier genes expression at this instance, which indicated the growth promotion was closely related to intestinal mucosal integrity. The present study showed that high dietary TDF could enhance the cecal mucosal integrity of ducks by increasing VH and MLT and lowering CD, and it was accompanied by increasing barrier gene expression of *ZO-1, Claudin-1, and Muc2* at high dietary TDF level. Our results were partly supported by Han et al. (2017), who observed that the growth promotion of starter Pekin ducks caused by high crude fiber was accompanied with increasing gizzard weight and improved jejunal morphology. Furthermore, when Pekin ducks were fed resistant starch-supplemented diets, the increasing MLT, VH, and V/C of ileum or cecum was followed by the markedly increasing genes expression of *ZO-1, Claudin-1*, and *Muc2* in these tissues (Qin et al., 2019, 2020). Tight junction proteins are contributed to the intercellular junctional complexes between intestinal epithelial cells, which sealed the paracellular space between these cells and regulated the permeability of the intestinal barrier (Ulluwishewa et al., 2011). Mucins are synthesized and secreted from goblet cells, and they are the major component of the mucus layer (Montagne et al., 2004; Johansson, 2014). All these proteins are contributed to intestinal mucosal integrity and played crucial roles in gastrointestinal protection. Therefore, the duck cecal histological development regulated by high dietary TDF may be mediated by increasing gene expression of tight junction protein and mucins at this instance.

The diversity in intestinal microbiota based on the amount of OTUs was altered by the levels of dietary fiber. There was conspicuously different in α-diversity among the three levels of dietary fiber groups. The estimators of ace and chao1 ameliorated with increasing dietary fiber levels, indicating that dietary fiber could positively modulate species richness. Shannon index value was higher in the 16.2% TDF group, in which species diversity was more blooming. In line with previous study, Qin et al. (2020) reported dietary fiber had beneficial effects on diversity of cecal microbial communities of ducks. With the abundant bacterial diversity in the poultry hindgut, competitive inhibition on pathogenic bacteria could be magnified (Zheng et al., 2019). Community structures of all groups were compared by PCoA analysis. The PCoA plot revealed cecal microbial communities were distributed in three detached clusters, which showed microbial composition was distinct among three levels of dietary fiber. Further, ANOSIM analysis confirmed the separate clusters in different content of dietary fiber groups were significantly dissimilar (*R*^2^ = 0.336, *P* < 0.05). Consequently, it is reasonable to modulate microbial profile in the hindgut of ducks with dietary fiber diet.

Dietary fiber could alter microbial flora to present its function of healthy facilitation. Microbiota in the poultry gut was regarded as a barrier to defend against disease by eliminating pathogens and enhancing the immune system (Oakley et al., 2014). In the present study, Firmicutes and Bacteroidota were still the dominant phyla and Firmicutes had higher relative abundance, though ducks were fed different levels of dietary fiber diet, which was in line with the study of Wei et al. (2013). The improvement in the relative abundance of Firmicutes could bring benefits to normal colonization of microbial communities. Mulder et al. (2009) indicated that diet supplemented with dietary fiber had a positive role in minimizing pathogens. In the current study, higher Firmicutes to Bacteroidota ratio was facilitated by dietary fiber. The raised abundance of family Oscillospiraceae and Lachnospiraceae at expense of abundance of family Bacteroidaceae and Selenomondaceae contributed to higher Firmicutes to Bacteroidota ratio. Molist et al. (2012) reported dietary fiber promoted higher Firmicutes to Bacteroidota ratio in colon of piglets resulting in the reduced pathogenic infection chance. Oscillospiraceae and Lachnospiraceae known as butyrate-producing bacteria fermented dietary fiber into butyrate to limit pathogenic bacteria colonization (Guilloteau et al., 2010; Meehan and Beiko, 2014). Moreover, the positive correlation between genus *Bacteroides* of family Bacteroidaceae and pro-inflammatory cytokines including IL-8 and TNF-α was reported by previous study (Wang et al., 2019). Therefore, the counterbalance of intestinal microbiota modulated by dietary fiber could be a biomarker to estimate intestinal lumen function of duck.

Admittedly, the changes in the relative abundance of microbiota in the gut ecosystem are pronouncedly linked with its metabolites. It was observed that dietary fiber as a substrate for microbial fermentation could be catabolized into SCFAs (Walugembe et al., 2015). It was established that the sequences representative of Firmicutes and Bacteroidota were major executors in the pathway of SCFAs metabolism, in which Firmicutes catabolized dietary fiber into propionate and butyrate while Bacteroidota specialized in producing propionate (Pandit et al., 2018). The relative abundance of Lachnospiraceae and Oscillopiraceae, butyrate producers (Biddle et al., 2013; Gophna et al., 2017), affiliating to Firmicutes significantly increased when ducks were fed higher levels of dietary fiber diet. Walugembe et al. (2015) reported that the relative abundance of butyrate producers like Ruminococcaceae was increased in the cecum of laying-hen chicks in the high dietary fiber group. Chen et al. (2020) also observed that dietary fiber could not only ameliorate the concentration of butyrate, but elevate the relative abundance of microbiota producing butyrate in the cecum of weaning piglets. In agreement with our study, the relative abundance of butyric-producing bacteria including Lachnospiraceae and Oscillopiraceae distinctly increased, by which Firmicutes abundance presented an ascent, and butyrate as its production also subsequently changed with an increase in TDF. Intriguingly, the expectedly augmented value of butyrate concentration as 14.7% TDF diet was lessening after supplying 16.2% TDF diet, which might be associated with the decline in the relative abundance of *Butyricicoccus* in the 16.2% TDF group. Since *Butyricicoccus*, pertaining to family Butyricicoccaceae, is a branch of producing-butyric bacteria colonizing the hindgut (Nava and Stappenbeck, 2011; Zvanych et al., 2014). However, the concentration of propionate was lowered with an increase in TDF that was in contrast to the findings of Qin et al. (2020), who observed that dietary fiber could ameliorate the concentration of propionate in the cecum. The difference could be partially explained by the lower abundance of *Megamonas* in the higher levels of dietary fiber groups in the present study given that *Megamonas* favored the pathway of propionate fermentation (Sergeant et al., 2014). Besides, *Bacteroides* presented a positive correlation with propionate from our observation corroborating the conclusion that *Bacteroides* were actively involved in the propionate fermentation pathway (Sergeant et al., 2014). The results illustrated microbial ecosystem plays a particular role in dietary fiber degradation and SCFAs production. Meanwhile, increasing dietary fiber has a distinct advantage in modulating microbial structure in the cecum of ducks.

Short-chain fatty acids were the major metabolites of dietary fiber fermentation by microbiota in the cecum of duck. Butyrate, one of the fermentable products, not only played a key role in energy salvage but improved the function of the intestinal barrier (Peng et al., 2009). Muc2 was a glycoprotein layer on the epithelial cell to structure mucus barrier, which was modulated by *Muc2* gene, and butyrate is beneficial to up-regulate the expression of *Muc2* gene (Smirnov et al., 2005). In the present study, the expression of *Muc2* gene was conspicuously raised in the diet containing 14.7% TDF with a higher concentration of butyrate. In line with our study, Wellington et al. (2020) reported that high dietary fiber treatment elevated butyrate concentration and *Muc2* gene expression in the cecum. Trachsel et al. (2018) revealed that dietary fiber diet containing 5% resistant potato starch ameliorated concentration of butyrate and stimulated the expression of *Muc2* gene in the cecum. Besides, tight junctions could decrease paracellular permeability to limit pathogens and antigens (Groschwitz and Hogan, 2009). The tight junctions were mainly comprised of claudins, occludins, and zonula occludins, which were positively stimulated by butyrate (Kim et al., 2012). The expression of *Claudin-1* and *ZO-1* genes in the cecum increased with higher concentration of butyrate after ducks being fed dietary fiber diets containing 14.7 and 16.2% TDF. The results were in accordance with the study of Chen et al. (2013), who revealed that dietary fiber could elevate butyrate concentration and the expression of *Claudin-1* and *ZO-1* genes in the ileum of piglets. Therefore, combined with the improvement of cecal morphology and barrier genes expressing at high dietary TDF, it could be speculated that increasing dietary TDF could produce more butyrate by altering intestinal microbiota to stimulate intestinal development in ducks.

## Conclusion

In a nutshell, increasing TDF levels could bring benefits to growth performance and intestinal health in meat ducks. Important biomarkers like elevated cecal histomorphology parameters, changed microbiota communities, enhanced SCFAs concentrations, and up-regulated expression of barrier-related genes were observed. Meanwhile, these results powerfully revealed that changes of cecal microbiota communities might conduce to the improvement of butyrate concentration, which subsequently exerted beneficial effects on the intestinal barrier function, facilitating the interaction between intestinal mucosa and microbiota.

## Data Availability Statement

The datasets presented in this study can be found in online repositories. The names of the repository/repositories and accession number(s) can be found below: https://www.ncbi.nlm.nih.gov/, PRJNA731120.

## Ethics Statement

The animal study was reviewed and approved by animal care and use committee of Institute of Animal Sciences of Chinese Academy of Agricultural Sciences.

## Author Contributions

YH implemented the study and wrote the manuscript. YH, ZS, ZJ, YW, BZ, and JT assisted in conducting the experiment and collecting samples. SH and MX designed the experiment. MX revised the manuscript. All authors contributed to the study and supported the submitted version.

## Conflict of Interest

The authors declare that the research was conducted in the absence of any commercial or financial relationships that could be construed as a potential conflict of interest.

## Publisher’s Note

All claims expressed in this article are solely those of the authors and do not necessarily represent those of their affiliated organizations, or those of the publisher, the editors and the reviewers. Any product that may be evaluated in this article, or claim that may be made by its manufacturer, is not guaranteed or endorsed by the publisher.

## References

[B1] AOAC International (2007). *Official Methods of Analysis of AOAC International.* Gaithersburg, MD: Oxford University Press.

[B2] Biddle A. Stewart L. Blanchard J. Leschine S. (2013). Untangling the genetic basis of fibrolytic specialization by *Lachnospiraceae* and ruminococcaceae in diverse gut communities. *Diversity* 3 627–640. 10.3390/d5030627

[B3] Chen T. Chen D. Tian G. Zheng P. Mao X. Yu J. (2020). Effects of soluble and insoluble dietary fiber supplementation on growth performance, nutrient digestibility, intestinal microbe and barrier function in weaning piglet. *Anim. Feed Sci. Technol.* 260:114335. 10.1016/j.anifeedsci.2019.114335

[B4] Chen H. Mao X. He J. Yu B. Huang Z. Yu J. (2013). Dietary fibre affects intestinal mucosal barrier function and regulates intestinal bacteria in weaning piglets. *Br. J. Nutr.* 10 1837–1848. 10.1017/S0007114513001293 23656640

[B5] Du X. Xiang Y. Lou F. Tu P. Zhang X. Hu X. (2020). Microbial community and short-chain fatty acid mapping in the intestinal tract of quail. *Animals* 10:1006. 10.3390/ani10061006 32526858 PMC7341218

[B6] Edgar R. C. (2010). Search and clustering orders of magnitude faster than BLAST. *Bioinformatics* 19 2460–2461. 10.1093/bioinformatics/btq461 20709691

[B7] Edgar R. C. Haas B. J. Clemente J. C. Quince C. Knight R. (2011). UCHIME improves sensitivity and speed of chimera detection. *Bioinformatics* 16 2194–2200. 10.1093/bioinformatics/btr381 21700674 PMC3150044

[B8] Gophna U. Konikoff T. Nielsen H. B. (2017). Oscillospira and related bacteria-From metagenomic species to metabolic features. *Environ. Microbiol.* 19 835–841. 10.1111/1462-2920.13658 28028921

[B9] Groschwitz K. R. Hogan S. P. (2009). Intestinal barrier function: molecular regulation and disease pathogenesis. *J. Allergy Clin. Immunol.* 124 3–20. 10.1016/j.jaci.2009.05.038 19560575 PMC4266989

[B10] Guilloteau P. Martin L. Eeckhaut V. Ducatelle R. Zabielski R. Van Immerseel F. (2010). From the gut to the peripheral tissues: the multiple effects of butyrate. *Nutr. Res. Rev.* 23 366–384. 10.1017/S0954422410000247 20937167

[B11] Han H. Y. Zhang K. Y. Ding X. M. Bai S. P. Luo Y. H. Wang J. P. (2017). Effect of dietary fiber levels on performance, gizzard development, intestinal morphology, and nutrient utilization in meat ducks from 1 to 21 days of age. *Poult. Sci.* 12 4333–4341. 10.3382/ps/pex268 29053831

[B12] Johansson M. E. (2014). Mucus layers in inflammatory bowel disease. *Inflamm. Bowel Dis.* 11 2124–2131. 10.1097/MIB.0000000000000117 25025717

[B13] Kheravii S. K. Morgan N. K. Swick R. A. Choct M. Wu S. B. (2018). Roles of dietary fibre and ingredient particle size in broiler nutrition. *Worlds Poult. Sci. J.* 74 301–316. 10.1017/s0043933918000259

[B14] Kim J. C. Hansen C. F. Mullan B. P. Pluske J. R. (2012). Nutrition and pathology of weaner pigs: nutritional strategies to support barrier function in the gastrointestinal tract. *Anim. Feed Sci. Technol.* 20 3–16. 10.1016/j.anifeedsci.2011.12.022

[B15] Magoc T. Salzberg S. L. (2011). FLASH: fast length adjustment of short reads to improve genome assemblies. *Bioinformatics* 21 2957–2963. 10.1093/bioinformatics/btr507 21903629 PMC3198573

[B16] Meehan C. J. Beiko R. G. (2014). A phylogenomic view of ecological specialization in the *Lachnospiraceae*, a family of digestive tract-associated bacteria. *Genome Biol. Evol.* 3 703–713. 10.1093/gbe/evu050 24625961 PMC3971600

[B17] Molist F. Manzanilla E. G. Perez J. F. Nyachoti C. M. (2012). Coarse, but not finely ground, dietary fibre increases intestinal firmicutes:bacteroidetes ratio and reduces diarrhoea induced by experimental infection in piglets. *Br. J. Nutr.* 1 9–15. 10.1017/S0007114511005216 22018207

[B18] Montagne L. Piel C. Lallès J. P. (2004). Effect of diet on mucin kinetics and composition: nutrition and health implications. *Nutr. Rev.* 3 105–114. 10.1301/nr.2004.mar.105-114 15098857

[B19] Mulder I. E. Schmidt B. Stokes C. R. Lewis M. Bailey M. Aminov R. I. (2009). Environmentally-acquired bacteria influence microbial diversity and natural innate immune responses at gut surfaces. *BMC Biol.* 7:79. 10.1186/1741-7007-7-79 19930542 PMC2785767

[B20] Nava G. M. Stappenbeck T. S. (2011). Diversity of the autochthonous colonic microbiota. *Gut Microbes* 2 99–104. 10.4161/gmic.2.2.15416 21694499 PMC3225773

[B21] Oakley B. B. Lillehoj H. S. Kogut M. H. Kim W. K. Maurer J. J. Pedroso A. (2014). The chicken gastrointestinal microbiome. *FEMS Microbiol. Lett.* 2 100–112. 10.1111/1574-6968.12608 25263745

[B22] Pandit R. J. Hinsu A. T. Patel N. V. Koringa P. G. Jakhesara S. J. Thakkar J. R. (2018). Microbial diversity and community composition of caecal microbiota in commercial and indigenous Indian chickens determined using 16s rDNA amplicon sequencing. *Microbiome* 6:115. 10.1186/s40168-018-0501-9 29935540 PMC6015460

[B23] Peng L. Y. Li Z. R. Green R. S. Holzman I. R. Lin J. (2009). Butyrate enhances the intestinal barrier by facilitating tight junction assembly via activation of AMP-activated protein kinase in Caco-2 cell monolayers. *J. Nutr.* 9 1619–1625. 10.3945/jn.109.104638 19625695 PMC2728689

[B24] Pu G. Li P. Du T. Niu Q. Fan L. Wang H. (2020). Adding appropriate fiber in diet increases diversity and metabolic capacity of distal gut microbiota without altering fiber digestibility and growth rate of finishing pig. *Front. Microbiol.* 11:533. 10.3389/fmicb.2020.00533 32328041 PMC7160236

[B25] Qin S. M. Zhang K. Y. Ding X. M. Bai S. P. Wang J. P. Zeng Q. F. (2019). Effect of dietary graded resistant potato starch levels on growth performance, plasma cytokines concentration, and intestinal health in meat ducks. *Poult. Sci.* 98 3523–3532. 10.3382/ps/pez186 31329991

[B26] Qin S. Han H. Zhang K. Ding X. Bai S. Wang J. (2018). Dietary fibre alleviates hepatic fat deposition via inhibiting lipogenic gene expression in meat ducks. *J. Anim. Physiol. Anim. Nutr.* 2 e736–e745. 10.1111/jpn.12828 29105186

[B27] Qin S. Zhang K. Applegate T. J. Ding X. Bai S. Luo Y. (2020). Dietary administration of resistant starch improved caecal barrier function by enhancing intestinal morphology and modulating microbiota composition in meat duck. *Br. J. Nutr.* 123 172–181. 10.1017/S0007114519002319 31495347

[B28] Sergeant M. J. Constantinidou C. Cogan T. A. Bedford M. R. Penn C. W. Pallen M. J. (2014). Extensive microbial and functional diversity within the chicken cecal microbiome. *PLoS One* 9:e91941. 10.1371/journal.pone.0091941 24657972 PMC3962364

[B29] Singh A. K. Kim W. K. (2021). Effects of dietary fiber on nutrients utilization and gut health of poultry: a review of challenges and opportunities. *Animals* 11:181. 10.3390/ani11010181 33466662 PMC7828824

[B30] Smirnov A. Perez R. Amit-Romach E. Sklan D. Uni Z. (2005). Mucin dynamics and microbial populations in chicken small intestine are changed by dietary probiotic and antibiotic growth promoter supplementation. *J. Nutr.* 135 187–192. 15671211 10.1093/jn/135.2.187

[B31] Tang D. Li Z. Mahmood T. Liu D. Hu Y. Guo Y. (2020). The association between microbial community and ileal gene expression on intestinal wall thickness alterations in chickens. *Poult. Sci.* 99 1847–1861. 10.1016/j.psj.2019.10.029 32241465 PMC7587722

[B32] Tejeda J. O. Kim W. K. (2021). Role of dietary fiber in poultry nutrition. *Animals* 11:461. 10.3390/ani11020461 33572459 PMC7916228

[B33] Tejeda O. J. Kim W. K. (2020). The effects of cellulose and soybean hulls as sources of dietary fiber on the growth performance, organ growth, gut histomorphology, and nutrient digestibility of broiler chickens. *Poult. Sci.* 99 6828–6836. 10.1016/j.psj.2020.08.081 33248598 PMC7704948

[B34] Trachsel J. Briggs C. Gabler N. K. Allen H. K. Loving C. L. (2018). Resistant potato starch fuels beneficial host-microbe interactions in the gut. *bioRxiv* 389007 [Preprint]. 10.1101/389007

[B35] Ulluwishewa D. Anderson R. C. McNabb W. C. Moughan P. J. Wells J. M. Roy N. C. (2011). Regulation of tight junction permeability by intestinal bacteria and dietary components. *J. Nutr.* 141 769–776. 10.3945/jn.110.135657 21430248

[B36] Walugembe M. Hsieh J. C. Koszewski N. J. Lamont S. J. Persia M. E. Rothschild M. F. (2015). Effects of dietary fiber on cecal short-chain fatty acid and cecal microbiota of broiler and laying-hen chicks. *Poult. Sci.* 10 2351–2359. 10.3382/ps/pev242 26316341

[B37] Wang W. W. Jia H. J. Zhang H. J. Wang J. Lv H. Y. Wu S. G. (2019). Supplemental plant extracts from flos lonicerae in combination with baikal skullcap attenuate intestinal disruption and modulate gut microbiota in laying hens challenged by *Salmonella* pullorum. *Front. Microbiol.* 10:1681. 10.3389/fmicb.2019.01681 31396190 PMC6668501

[B38] Wei S. Morrison M. Yu Z. (2013). Bacterial census of poultry intestinal microbiome. *Poult. Sci.* 92 671–683. 10.3382/ps.2012-02822 23436518

[B39] Wellington M. O. Thiessen R. B. Van Kessel A. G. Columbus D. A. (2020). Intestinal health and threonine requirement of growing pigs fed diets containing high dietary fibre and fermentable protein. *Animals* 11:2055. 10.3390/ani10112055 33171958 PMC7694666

[B40] Zheng M. Mao P. Tian X. Guo Q. Meng L. (2019). Effects of dietary supplementation of alfalfa meal on growth performance, carcass characteristics, meat and egg quality, and intestinal microbiota in Beijing-you chicken. *Poult. Sci.* 98 2250–2259. 10.3382/ps/pey550 30496504

[B41] Zvanych R. Lukenda N. Kim J. J. Li X. Petrof E. O. Khan W. I. (2014). Small molecule immunomodulins from cultures of the human microbiome member *Lactobacillus plantarum*. *J. Antibiot.* 67 85–88. 10.1038/ja.2013.126 24281660

