# Supplementary material for: Effects of Total Dietary Fiber on Cecal Microbial Community and Intestinal Morphology of Growing White Pekin Duck
Source: Front Microbiol. 2021 Sep 1;12:727200. doi: 10.3389/fmicb.2021.727200 (PMC8440899; doi:10.3389/fmicb.2021.727200)
Supplement: Supplementary file 1 [file Table_1.DOCX]

**Supplementary Table S1.** The primers for quantitative real-time PCR

| Gene | Gene ID | Primer | Sequence (5′-3′) | Size (bp) |
| --- | --- | --- | --- | --- |
| *ZO-1* | XM_038184905.1 | Forward | gagccttcagaccattccagaca | 155 |
|  |  | Reverse | tcgcctgccacctcttccata |  |
| *MUC2* | XM_038180256.1 | Forward | ccataagccagaccacgccatc | 143 |
|  |  | Reverse | caaggtgctcaaggtgctccag |  |
| *Occludin* | XM_013109403.1 | Forward | caggatgtggcagaggaatacaa | 160 |
|  |  | Reverse | ccttgtcgtagtcgctcaccat |  |
| *Claudin 1* | XM_013108556.4 | Forward | gaccaggtgaagaagatgcggatg | 107 |
|  |  | Reverse | cgagccactctgttgccatacc |  |
| *β-actin* | NM_001310408.1 | Forward | ccagccatctttcttgggta | 105 |
|  |  | Reverse | gtgttggcgtacaggtcctt |  |

Muc2, Mucin-2; ZO-1, Zonula occludens-1.
